# Mechanisms of Chromium Removal from Water and Soil Using Bioleached Nano Zero-Valent Iron-Mediated Biochar via Co-Pyrolysis

**DOI:** 10.3390/nano14231895

**Published:** 2024-11-26

**Authors:** Zhiyi Liu, Shuhong Zhou, Yubing Cai, Xuehai Zhang, Muhammad Shaaban, Qi-an Peng, Yajun Cai

**Affiliations:** 1 School of Resources and Environment, Wuhan Textile University, Wuhan 430200, China; 2 China National Grain Reserve Management Group Corporation Anhui Branch Lu’an Depot, Lu’an 237000, China; 3 College of Agriculture, Henan University of Science and Technology, Luoyang 471000, China; shabanbzu@hotmail.com; 4 Clean Production of Textile Printing and Dyeing Engineering Research Center, Ministry of Education, Wuhan 430200, China

**Keywords:** bioleaching, Cr, biochar-loaded nano zero-valent iron, microorganisms

## Abstract

Biological charcoal loaded with nano zero-valent iron (nZVI@BC) was synthesized using the bioleaching co-pyrolysis method. This study analyzed the formulation sequence of nZVI@BC and its influence on chromium elimination from water and soil, along with the involved mechanisms. The bioleaching method facilitated ionic iron incorporation onto biochar in the form of yellow potassium ferroalum compounds, which were reduced to Fe^0^ by H_2_, CO, and CH_4_ generated during biomass co-pyrolysis. In aqueous conditions, the removal capacity of Cr(VI) by nZVI@BC increased by 72.01% and 66.92% compared to biochar (BC) and biochar–bioleachate composite (BBC), respectively. Under optimal conditions, nZVI@BC eliminated 90.11% of 20 mg/L Cr(VI), with experimental data fitting the Freundlich and pseudo-second-order kinetic models. The nZVI@BC also showed a passivation effect on chromium in soil; after 45 days, the exchangeable state of chromium was reduced by 12.89%, while the residual state increased by 10.45%. This enhancement in chromium elimination from soil was evident, as the residual state increased more for nZVI@BC (10.45%) than for BC alone (9.67% and 8.48%). Soil physicochemical properties and microbial community abundance improved as well. Cr(VI) removal mechanisms involved adsorption, reduction, and co-precipitation in water, while soil mechanisms included surface adsorption, electrostatic attraction, ion exchange, and complexation. The synthesis of nZVI@BC offers a novel method for creating iron-modified materials to effectively remove Cr(VI).

## 1. Introduction

The heavy metal chromium is one of the three internationally acknowledged carcinogens, adversely affecting plants, aquatic animals, and microorganisms. With the development of the electroplating, printing and dyeing, and metal processing industries, chromium pollution in water bodies and soil has significantly increased [1]. Common methods for removing chromium from water bodies include chemical precipitation, ion exchange, and adsorption, while methods for removing chromium from soil encompass chemical stabilization remediation, chemical drenching, and bioremediation [2]. Among these methods, adsorption and chemical stabilization remediation are more straightforward to implement, more cost-effective, and demonstrate superior remediation effects than the most commonly employed techniques for removal of Cr(Ⅵ). Biochar (BC) is an effective adsorbent and passivator characterized by a wide range of sources and low cost. Its well-developed pore structure, generous specific surface area, and abundant functional groups enable efficient adsorption of heavy metals in water and soil [3]. The addition of BC can significantly enhance soil fertility and regulate the abundance and activity of soil microorganisms [4]. Nano-zero-valent iron (nZVI) consists of iron particles with sizes ranging from 1 to 100 nm, possessing a large specific surface area, high reactivity, and reducing properties. It is also a commonly used soil passivator with promising prospects in environmental remediation. However, nZVI is prone to oxidation and agglomeration when exposed to air. To address these challenges, researchers have conducted extensive studies on the preparation of biochar and nZVI composites. The preparation methods for nZVI@BC mainly include liquid phase reduction, green synthesis, and co-pyrolysis [5]. Among these methods, co-pyrolysis involves impregnating biomass with a solution of iron salts or iron compounds, drying it, and then calcining the iron-loaded biomass under high-temperature anaerobic conditions. The iron source catalyzes the cleavage of biomass polymers, producing small molecules of reducing gases such as CO, CH_4_, and H_2_. Simultaneously, these gases can reduce iron to both divalent and zero-valent forms [6]. This method offers advantages such as low raw material cost, elimination of the need for additional reducing agents, and ease of scaling up for continuous production [7]. However, the preparation process of iron-loaded biochar can be energy-intensive and may have low raw material utilization. Thus, there is a pressing need to establish a greener and more efficient method for creating iron-loaded biochar. Bioleaching, also known as biological hydrometallurgy technology [8], is based on the principle that bacteria oxidatively reduce iron or sulfur in the ore, releasing the metals in the ore into water while utilizing the energy necessary for growth [9]. The present study builds upon this foundation, utilizing biochar and microorganisms to prepare iron-loaded biochar.

The purpose of this research is to explore a novel method for preparing nZVI@BC by integrating bioleaching technology with co-pyrolysis. Additionally, it investigates the removal efficiency and mechanisms of Cr(Ⅵ) in water and soil, as well as examines the impact of the material on the abundance of soil microbial communities.

## 2. Material and Methods

### 2.1. Material Preparation

The *Thiobacillus ferrooxidans* used in this experiment was isolated from activated sludge in the laboratory and designated *Acidithiobacillus ferrooxidans* FD319B (*Acidithiobacillus ferrooxidans*). The BC was obtained from agricultural straw and wood gasification by-products in Tongshan County, Hubei Province. It was dried and ground indoors, then separated using a 60-mesh fine sieve. The experimental soil was sourced from rice fields in Jingmen City, Hubei Province, and characterized as brown loam. The soil was collected while removing stones, weeds, and other debris, then naturally air-dried before being ground and sieved. Iron-carrying precursors (BBC) were derived by adding both Thiobacillus ferrooxidans and biochar to a modified 9K medium following 72 h of bioleaching, as described by [10]. The BC was placed in the nitrogen inlet of the tube furnace, while the BBC was positioned in the middle, then pyrolyzed at 1000 °C with a heating rate of 10 °C/min and a holding time of 1 h to produce nZVI@BC, referred to as C-PBC1000. Additionally, a pyrolysis control group was established, wherein only the BBC was placed in the tube furnace, and the biochar was pyrolyzed at 600, 800, and 1000 °C. The resulting biochars were designated as PBC600, PBC800, and PBC1000, respectively.

### 2.2. Material Analysis

The thermogravimetric analysis of BBC was carried out using thermogravimetry (TG, Rigaku, Tokyo, Japan) to investigate the physical transformation during the pyrolysis of BBC to PBC. The morphological characteristics of BC, BBC, PBC, and nZVI@BC were observed using scanning electron microscopy (SEM, Regulus8100 Hitachi, Ltd, Tokyo, Japan). PBC and nZVI@BC were characterized by X-ray diffractometry (XRD, D8 Brukr Adv., Oberkochen, Germany), which determined the surface compositions of the materials and nanomaterials at different temperatures. The presence of functional groups of BC, BBC, and nZVI@BC was analyzed using Fourier transform infrared spectroscopy (FTIR, Nexus 670, Thermo Nicolet , Ltd, Madison, MA, USA). The comparative surface area and pore structures of BC, BBC, and nZVI@BC were assessed using a fully automated specific surface area and pore size analyzer (BET, Micromeritics 3Flex, Quanta chrome Instruments, Boynton, FL, USA). Finally, the elemental compositions and valence states of the materials before and after the adsorption were evaluated using X-ray photoelectron spectroscopy (XPS, Therm. Scient. K-Alpha, Boston, MA, USA).

### 2.3. Research Content

The removal capabilities of BC, BBC, PBC, and nZVI@BC for chromium in water bodies were compared. The impact of temperature, pH, and other physicochemical conditions on the elimination of Cr(VI) was investigated, and coexisting ions were incorporated to examine the removal efficiency of nZVI@BC in real water bodies. The remediation effects of different materials, dosages, and remediation times on Cr(VI)-contaminated soils were assessed. Additionally, the relationship between the materials and pH value, as well as the chromium fugitive morphology, was explored. The diversity and abundance of microorganisms in heavy-metal-contaminated soils were analyzed.

### 2.4. Experimental Methods

The determination of Cr(VI) solution was conducted following “Determination of Hexavalent Chromium in Water Quality by Dibenzoyl Dihydrazine Spectrophotometry”. Tessier’s five-step sequential extraction procedure (SPE) was utilized to extract chromium from soil, allowing for the analysis of chromium forms in exogenously contaminated soils. Ultrapure water was employed as the extraction solution, and the pH of the soil was measured using a glass electrode meter in a 1:5 mixture of soil and ultrapure water. The primers used for high-throughput sequencing included a universal primer (5′ACTCCTACGGGGAGGCAGCA-3′) and a reverse primer (5′GGACTACHVGGGTWTCTAAT-3′). Fluorescence PCR was performed using the SYBR Green method. Table S1 provides the primer information for the sequenced samples.

## 3. Results

### 3.1. Bioleaching Process

**Figure 1:**
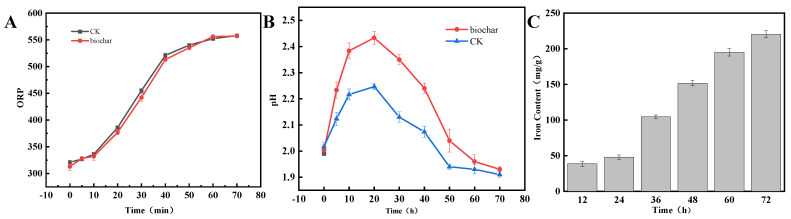
Variation of ORP (**A**), pH (**B**), and iron content of biochar (**C**) during the bioleaching process.

The ORP value reached 552 at 70 h of incubation, and the change in ORP following the addition of BC remained essentially unchanged compared to the blank group (CK) (Figure 1A). *Thiobacillus oxidans* exhibited a significant pH change during its growth process (Figure 1B), generally displaying an upward trend followed by a downward trend. In comparison to the blank control group, the addition of biochar resulted in a relatively large increase in pH during the pre-growth phase. During the bioleaching process, the iron content in biochar increased, with longer incubation times (Figure 1C) reaching up to 217.5 mg/g after 72 h of incubation.

### 3.2. Characterization

**Figure 2:**
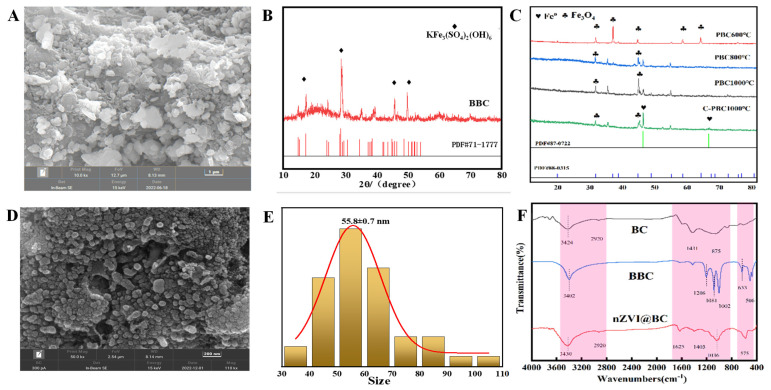
SEM image of BBC (1 um) (**A**); XRD of BBC (**B**); XRD of PBC600, PBC800, and PBC1000 (**C**); SEM of nZVI@BC (200 nm) (**D**); average size of Fe_3_O_4_ crystal grain size on nZVI@BC (**E**); FTIR image of nZVI@BC, BBC, and BC (**F**).

The SEM image reveals that the surface of the BC was relatively smooth (Figure S1A) and featured pores of varying sizes at the micrometer level. In contrast, the surface of the BBC was densely populated with numerous irregular crystalline particles (Figure 2A), with particle sizes ranging from 0.5 to 1 μm. Furthermore, the XRD pattern of the BBC aligned with the standard card for KFe_3_(SO_4_)_2_(OH)_6_, indicating that the crystalline particles loaded on to the surface of the biochar were KFe_3_(SO_4_)_2_(OH)_6_ (Figure 2B). The XRD patterns (Figure 2C) of PBC600, PBC800, and PBC1000 show distinct Fe_3_O_4_ peaks, which gradually strengthen with increasing temperature. However, compared to PBC1000, the Fe_3_O_4_ content in C–PBC1000 decreased, and a new substance, Fe0, emerged. 

From the thermogravimetric curve of the BBC (Figure S1B), it is evident that the process of generating PBC occurred in three stages at temperatures of 390 °C and 785 °C. During co-pyrolysis of the BBC with sawdust in a tube furnace at 1000 °C, gas chromatography analysis of samples taken from the furnace revealed a significant increase in the percentage of reducing gases in the control group after the addition of sawdust. Specifically, the percentage of H_2_ increased from 14.07% to 22.15%, the percentage of CO rose from 29.44% to 51.42%, while the percentage of CH_4_ decreased from 6.97% to 0.89% (Table S2). Furthermore, the SEM images of PBC600 (Figure S2A), PBC800 (Figure S2C), and PBC1000 (Figure 2D) reveal that the particle size of the surface-loaded Fe_3_O_4_ crystals on biochar decreased significantly with increasing pyrolysis temperature. The Fe_3_O_4_ crystals on PBC1000 completely covered the surface, forming spherical particles with smaller particle sizes and exhibiting a more regular crystal structure. The results were analyzed using Nano Measurer software (ImageJ 1.8.0), which provided the average sizes of the crystal particles for PBC600, PBC800, and C–PBC1000 as 471.1 ± 9.3 nm (Figure S2B), 170.1 ± 3.6 nm (Figure S2D), and 55.8 ± 0.7 nm (Figure 2E), respectively. Scheduled surface functional groups of BC, BBC, and nZVI@BC, as determined by Fourier infrared spectroscopy (FTIR), are shown in Figure 2F. The functional groups on the surface of BC primarily consisted of hydroxyl (O–H), carbonyl (C=O), and C=C groups. The characteristic peak appearing at 3419 cm^−1^ corresponds to the O-H stretching vibration, while the peaks at 2920 cm^−1^, 1431 cm^−1^, and 875 cm^−1^ are attributed to the stretching vibrations of aromatic C=O, C=C, and C-H, respectively. The surface functional groups of BBC underwent significant changes, with a substantial increase in C-O groups occurring between 1000 cm⁻¹ and 1500 cm⁻¹. The nZVI@BC displayed typical Fe peaks, with the characteristic peaks at 575 cm^−1^ and 1036 cm^−1^ attributed to the stretching vibrations of Fe–O.

The changes in specific surface area, pore volume, and pore size of BC, BBC, and nZVI@BC are evident from the BET results (Table S3). Compared to BC (41.09 m^2^/g) and BBC (52.74 m^2^/g), the specific surface area of nZVI@BC significantly increased to 318.11 m^2^/g. According to IUPAC isothermal modelling classification (Figure S3), nZVI@BC now exhibited a type IV curve, with pore sizes primarily distributed in the range of 2–25 nm.

XPS analysis revealed the changes in surface elements before and after the adsorption of Cr (VI) on nZVI@BC (Figure S4A). The full-wave scanning spectra exhibited Cr2p signals, indicating successful adsorption of Cr. Element C was predominantly present in the forms of C–C, C–O, and C=O (Figure S4C), with peaks appearing at 284.2 eV, 286.3 eV, and 288.5 eV, respectively. Analysis of the Fe2p spectra (Figure S4E) showed an increase in the Fe atomic ratio; however, the Fe^0^ peak disappeared after the reaction, and Fe(III) and Fe(II) peaks emerged. The peaks at 576.4 eV and 587.6 eV, shown in Figure S4B, correspond to Cr(III) on the Cr2p orbital.

### 3.3. Results of Batch Experiments

**Figure 3:**
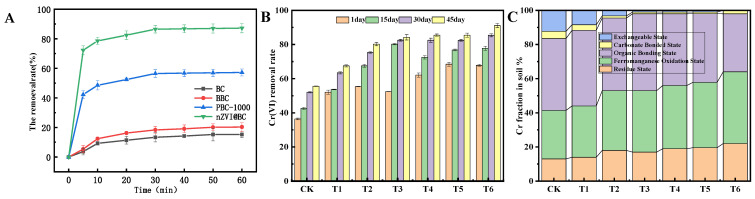
Remediation effects of different materials (**A**) with different dosages (**B**) on Cr(VI)-contaminated soil; morphology of Cr in soil after CK, BC, and nZVI@BC treatments (**C**).

Comparing the removal performances of BC, BBC, PBC-1000, and nZVI@BC (Figure 3A) clearly demonstrates that nZVI@BC achieved the highest removal of Cr(VI), reaching 87.23% in 60 min. PBC-1000 also showed a notable removal efficiency for Cr(VI), with a removal rate of 57.21% in the same timeframe. In contrast, BC and BBC exhibited poor removal performances, achieving only 15.22% and 20.31% removal of Cr(VI) in 60 min, respectively. A relatively low initial pH favored extraction of Cr(VI) from the water column, which gradually decreased as the pH increased (Figure S5A). The elimination of Cr(VI) reached 90.11% in 60 min at a pH of 3. However, when the pH was raised to 7, the removal efficiency decreased to 60.36%. At a pH of 11, a significant decrease in the removal was observed, with only 45.39% removal achieved in 60 min. The clearance of Cr(VI) by nZVI@BC decreased with increasing Cr(VI) concentration (Figure S5B). The elimination of Cr(VI) increased from 70.83% to 97.25% when the material dosage increased from 0.1 g/L to 0.9 g/L. Cr(VI) was nearly completely removed when the initial concentration was set between 10 and 50 mg/L (Figure S5C), with a removal rate of 99.54% achieved within 60 min at an initial concentration of Cr(VI) 10 mg/L. However, when the initial concentration increased to 50 mg/L, the removal rate decreased to 79.25%. Additionally, as the temperature increased from 15 °C to 35 °C (Figure S5D), the elimination of Cr(VI) increased from 90.85% to 97.13% within the same time. The presence of anions such as SO_4_^2−^, Cl^−^, and NO_3_^−^ in natural water bodies can compete with Cr(VI) for adsorption sites, thereby affecting the removal efficiency of Cr(VI) by nZVI@BC. As shown in Figure 3C, the elimination of Cr(VI) in the presence of SO_4_^2−^, Cl^−^, and NO_3_^−^, showed varying degrees of change over a 60 min period (Figure S5E). Notably, Cl^−^ had a greater impact, resulting in a decrease in removal efficiency from 99.27% to 93.85%.

The proposed primary (Figure S6A) and secondary (Figure S6B) kinetic models were applied to the data on Cr(VI) adsorption by BC, BBC, PBC, and nZVI@BC aligned more closely with the fitted secondary kinetic model, yielding R^2^ values of 0.9709, 0.9932, 0.9996, and 0.9998, respectively. These values significantly exceed the primary kinetic model’s R^2^ values (0.9505, 0.9599, 0.9562, and 0.9574) (Table S4). The results indicate that the removal of Cr(VI) was dominated by chemisorption.

### 3.4. The nZVI@BC Remediation of Chromium-Contaminated Soil

Figure 3A,B shows the remediation effects of diverse passivation materials at different dosages on Cr(VI)-impregnated soil. The addition of 10 g/kg of BC (T1) displayed a certain level of remediation, achieving a Cr(VI) removal rate of 55.61% by the 45th day, which was 11.92% higher than the blank group (CK) that did not receive any remediation agent. Different gradients of nZVI@BC (T2-T6) were added to the contaminated soil. The results showed that the stabilization of heavy metals improved with increased doses of the remediation agents. Particularly, the elimination rates of Cr(VI) after 45 days of remediation reached 80.21%, 84.26%, 85.23%, 86.21%, and 91.12%, respectively.

**Figure 4:**
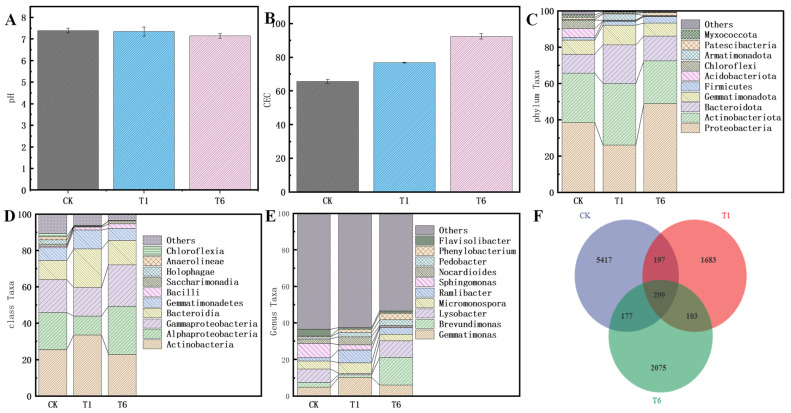
Changes in pH (**A**), CEC (**B**), and relative abundance of soil microorganisms on phylum (**C**), phylum (**D**), and genus (**E**) between treatments, and Venn diagram (**F**).

**Figure 5:**
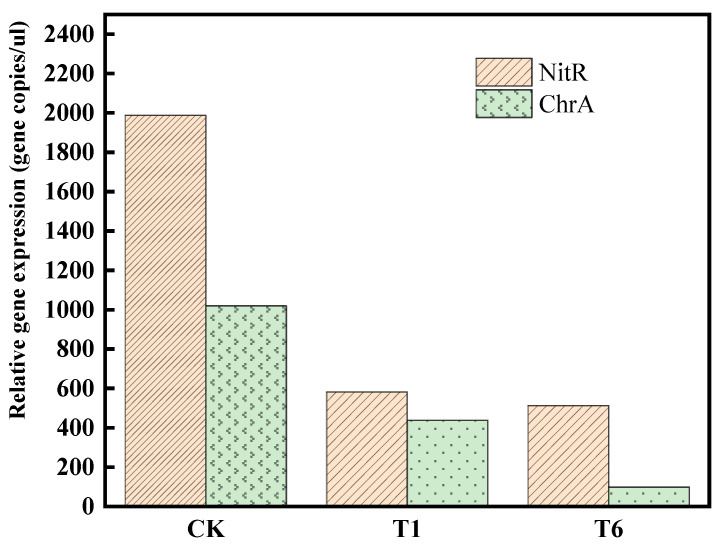
Results of fluorescence-quantitative PCR with different treatments.

The continuous extraction method was used to investigate the changes in the proportions of Cr among the morphological states following treatment with CK, BC, and nZVI@BC. In the CK group, 12.31% of Cr was found in the exchangeable state, 4.21% in the carbonate-bound state, 42.16% in the organic-bound state, 28.41% in the ferromanganese-oxidized state, and 13.01% in the residual state (Figure 3C). After treatment with BC in the T1 group, notable changes occurred in the distribution of Cr. Specifically, the exchangeable state of soil decreased by 3.89%, while the organically bound state increased by 2.14% compared to the CK group. In the T2–T6 groups, it was observed that with increasing doses of nZVI@BC, the proportions of exchangeable, organic-bound, and carbonate-bound Cr states in the soil gradually decreased, while the proportion of Fe-Mn oxidized state increased by 13.59% and the residual state increased by 8.91% compared to the CK group. After the restoration experiment, the pH levels of the soil for the CK, T1, and T6 groups were 7.39, 7.38, and 7.15, respectively (Figure 4A). The soil cation exchange capacity (CEC) values for the CK, T1, and T6 groups were 65.74 cmol^+^/kg, 76.81/Kg, and 92.46/Kg, respectively (Figure 4B). Alpha diversity index metrics are summarized in Table S5. The data in the table indicate a decrease in both Chao1 and Shannon values following the addition of nZVI@BC and BC. Chao1 values were reduced from 3746 to 1429 and T6 to 1763 after BC restoration, while Shannon values for BC were reduced from 8.91 to 6.78 and T6 to 7.09, respectively. The distribution changes of diverse microbial communities in chromium-contaminated soils at the phylum level can be seen in Figure 4C. The dominant phyla in the three treatment groups, i.e., CK, BC, and nZVI@BC, mainly comprised Proteobacteria, Actinobacteria, Bacteroidota, Gemmatimonadota, and Firmicutes. In the BC group, Proteobacteria decreased from 38.51% to 26.16% compared to CK, but increased to 49.05% after treatment with nZVI@BC. This indicates that nZVI@BC did not adversely affect microbial ecology and, to some extent, increased the abundance of metal-resistant bacteria. Figure 4D shows the changes in relative abundance at the phyla level, highlighting that Actinobacteria, α-Alphaproteobacteria, γ-Alphaproteobacteria, Bacteroidia, and Gemmatimonadetes are the dominant phyla shared by the three groups. All of these phyla consist of bacteria known for their chromium-reducing functions [11]. In group T1, the relative abundance of Actinobacteria increased from 25.52% to 33.48% compared to group CK. Conversely, alpha-proteobacteria decreased from 20.31% to 10.52%, while Bacteroidia increased from 10.49% to 21.21%. When compared to group T6, the abundance of Actinobacteria slightly decreased to 22.99%; however, alpha-proteobacteria (26.41%), gamma-proteobacteria (22.62%), and Bacteroidia (13.47%) all showed significant increases. Changes at the genus level were more pronounced in the treatment groups (Figure 4E). The dominant genera in the CK group were Sphingomonas (7.75%), Lysobacter (7.23%), Gemmatimonas (4.82%), Micromonospora 4.42%), Flavisolibacter (3.69%), Brevundimonas (2.71%), and Nocardioides (2.46%). The abundance of Sphingomonas decreased from 7.75% to 2.77% in the BC group compared to the CK group, while Gemmatimonas appeared as the dominant genus, with its relative abundance increasing to 10.21%. Micromonospora also increased to 5.77%, which is characteristic of metal-reducing bacteria. Brevundimonas increased to 15.21% in the T6 group, demonstrating absolute dominance. The composition and similarity of OTUs, along with their overlap among the three treatment groups, were analyzed using a Venn diagram (Figure 4F). This diagram visually compares the number of species that occur in common as well as those that are unique to each group. The total number of OTUs in the three groups of samples, CK, T1, and T6, were 6090, 2282, and 2654, respectively. Among these, there were 299 OTUs shared in common, while the number of unique OTUs for each group was 5417, 1683, and 2075, respectively. Compared to the CK group, the expression levels of NitR and ChrA were significantly decreased after BC and nZVI@BC were added (Figure 5). Notably, nZVI@BC showed a further decrease in the expression of chromium-associated resistance genes compared to BC.

## 4. Discussion

### 4.1. Preparation Process and Reaction Mechanism of nZVI@BC

ORP can indicate the relative content of Fe^2+^ and Fe^3+^ in the medium. A higher ORP value signifies a higher degree of conversion from Fe^2+^ to Fe^3+^. At 72 h, the higher ORP value indicates that Fe^2+^ is largely converted to Fe^3+^, and the addition of biochar does not affect the growth of the bacteria during the whole process. During the bioleaching process, sulfurous bacillus ferrous oxide oxidizes Fe^2+^ using H^+^ in the solution during the early stages of growth following a period of cultivation, and the hydrolysis reaction of the resulting Fe^3+^ causes the pH to decrease again. As a result, the pH initially rises and then subsequently falls [12].

The SEM and XRD results of BBC proved the existence of yellow potassium iron alum, and the C-O peaks observed in the FTIR spectrum of BBC resulted from *Thiobacillus ferrooxidans* producing a large amount of EPS on the surface of the biochar, further demonstrating the bacteria’s involvement in the material’s generation [13]. Based on the medium formulation and the bacterial growth process, the equation for the production of yellow potassium iron alum during the preparation of the material can be expressed as [14]:

4Fe^2+^ + O_2_ + 4H^+^ = 4Fe^3+^ + 2H_2_O (A. Involvement of ferrooxidans) (1)

K^+^ + 4Fe^3+^ + 2SO_4_^2−^+6H_2_O = KFe_3_ (SO_4_)_2_(OH)_6_ + 6H^+^ (2)

The presence of Fe_3_O_4_ confirmed by the XRD patterns of PBC600, PBC800, and PBC1000, with its content increasing alongside the pyrolysis at 1000 °C, was chosen. The GT data revealed the physical phase transformation during the preparation of PBC, which can be categorized into three stages: 1. First Stage (Equation (3)): 0–390 °C, during which the yellow potassium ferroalum crystals are destroyed. 2. Second Stage (Equation (4)): 390–785 °C, where the reaction products consist of iron oxide and sulphur trioxide. 3. Third Stage (Equation (5)): In this stage, some of the iron oxide material is reduced to triiron tetraoxide [15]. Additionally, the evaporation of water from the biochar and yellow potassium iron alum during the first stage, along with the desulphurization process in the second stage, contributes to the mass loss observed in the BBC.

K_2_Fe_6_(SO_4_)_4_(OH)_12_ = K_2_SO_4_Fe_2_(SO_4_)_3_ + Fe_2_O_3_ + 6H_2_O (3)

K_2_SO_4_∙Fe_2_(SO_4_)_3_ = Na_2_SO_4_ + Fe_2_O_3_ + 3SO_3_ (4)

6Fe_2_O_3_ + C = 4Fe_3_O_4_ + CO_2_ (5)

During the pyrolysis of C-PBC1000, the large amount of lignin present in the sawdust generates a large quantity of reducing gases, such as H_2_, CO and C_X_H_Y_ at high temperatures. These gases can reduce Fe_3_O_4_ to Fe_0_, explaining the presence of Fe_0_ in the characterization of C-PBC1000 and demonstrating the feasibility of the method. The possible reaction equations involved are as follows [16]:

3Fe_2_O_3_ + (H_2_,C,CO,C_X_H_Y_) → 2Fe_3_O_4_ + (H_2_O,CO,CO_2_,C_X_H_Y_O_Z_) (6)

Fe_3_O_4_ + 4H_2_ →3Fe^0^ + H_2_O (7)

Fe_2_O_3_ + 3H_2_→3Fe^0^ + H_2_O (8)

The decrease in crystal particle size with increasing pyrolysis temperature is likely due to the development of the internal pore structure of biochar, which creates more active sites and limits the growth of crystal particles. Simultaneously, higher temperatures facilitate the nucleation and growth of iron oxides, resulting in a decrease in the diameter of the crystal particles on the surface of the biochar [17]. The average crystal particle size of the C-PBC1000, approximately 55.8 nm, further demonstrates the successful preparation of nanoscale materials. The BET results indicate that the main pore type of nZVI@BC is a mesoporous structure, which offers substantial active sites [18]. Therefore, the experimentally prepared nZVI@BC exhibits an excellent specific surface area and porous structure, enhancing its ability to promote the adsorption of pollutants on its surface. The analysis of the XPS results revealed a significant change in the content of elemental C before and after adsorption, indicating that the surface functional groups of BC played a role in the elimination of Cr(VI). Related studies have shown that the hydroxyl and carboxyl groups on biochar could react with Cr to a certain extent [19], which is initially believed to involve both complexation and reduction reactions. Changes in the elements of Fe and Cr indicate that Fe_0_ participated in the reaction, facilitating a redox reaction with Cr(VI) [20]. The mechanism by which nZVI@BC removes Cr(VI) from water bodies can be inferred from the analysis above, as follows: 1. Adsorption: nZVI@BC adsorbs Cr(VI) onto the material’s surface through the porous structure of BC and the oxidized layer of nZVI. 2. Reduction: The Cr(VI) adsorbed on the nZVI@BC surface is reduced to Cr(III) by Fe^0^ and Fe^2+^, while a minor quantity of oxygen-containing functional groups also act as electron donors, facilitating the reduction of Cr(VI) to Cr(III). 3. Co-precipitation: The Cr(III) formed through reduction combines with OH- and precipitates as Cr(III)-Fe(III) hydroxide or co-precipitates as Cr_2_O_3_ [21].

### 4.2. Physicochemical Factors in the Removal of Hexavalent Chromium

Shahid et al. [22] showed that pH significantly affects the morphology of Cr(VI) in solution. For a pH range of 1.0 to 6.0, Cr(VI) primarily exists in the form of HCrO_4_^−^; at pH levels above 6.0, it mainly exists in the form of CrO_4_^2−^. Under acidic conditions, Cr(VI) reduction to Cr(III) is promoted, and the acidic environment can dissolve the passivation layer on the surface of zero-valent iron. Additionally, it effectively inhibits the formation of hydroxides and ferrochrome oxides, preventing these oxides from hindering the reduction of chromium by zero-valent iron [23]. In contrast, under alkaline conditions, the formation of hydroxides and ferrochrome oxides can hinder the reduction of chromium and inhibit electron transfer efficiency. The removal rate of nZVI@BC can be significantly improved by increasing the BC dosage. However, the percentage removal decreases with an increase in the initial concentration of Cr(VI). This decline occurs because a higher initial concentration leads to the occupation of active sites on the biochar, and the resulting passivation layer obstructs electron transfer, ultimately reducing the percentage removal [24]. Temperature is also a critical factor affecting the removal rate; higher system temperatures enhance the diffusion of Cr ions in the water column. The reaction is endothermic, and the increase in temperature facilitates this process [25]. Coupled with the zeta potential results (Figure S6F), it can be inferred that the surface of the carbon material carries a strong positive charge at low pH levels, facilitating the adsorption of anions. Therefore, in the presence of co-existing anions in the water, there will be competition for adsorption on the positively charged surface of nZVI@BC. This competition results in a decreased removal efficiency of Cr(VI).

In addition, in practical water applications, it is necessary to consider that anions such as SO_4_^2−^, Cl^−^, and NO_3_^−^ will compete with Cr(VI) for adsorption sites, thus affecting the removal efficiency of Cr(VI) by nZVI@BC.

### 4.3. Effect of nZVI@BC on Cr(VI) Removal and Microbial Abundance in Soils

In comparison to BC, nZVI@BC demonstrated a greater ability to effectively passivate Cr(VI) in soil and provide more reactive active sites for the curing reaction, particularly as the amount added increased [26], thereby improving the remediation efficiency. Fluorescence quantitative PCR technology was utilized to analyze the effects of different treatment groups on the expression levels of chromium reduction genes (NitR) and chromium resistance genes (ChrA) [27]. The observed decrease in chromium-related resistance genes in the nZVI@BC group compared to the BC group further confirms that nZVI@BC can effectively passivate Cr(VI) in soil. The level of soil heavy metal contamination is not only relevant to the total volume of heavy metals but is also closely linked to their accumulation patterns [28]. These patterns determine the process of heavy metal migration and transformation, ultimately affecting their bioavailability [29]. The nZVI@BC possesses a significant number of aerobic fluorophores on its membrane surface, allowing it to complex with trivalent chromium to form an organic bonding state. Consequently, chromium in the soil interacts with the iron oxides and iron hydroxides in the remediation agent, leading to complexation and precipitation, which results in the formation of Cr(III)/Fe(III) oxides or hydroxides [30]. Therefore, the biologically synthesized nZVI@BC is for effectively remediating Cr-polluted soils and transforming more toxic forms of chromium into harmless compounds for humans.

Soil pH can significantly affect the effectiveness of heavy metals in soil [31], while CEC measures the adsorption capacity of soil for various cations. CEC serves as an important index for evaluating soil fertility and can also reflect the stability of the soil to a certain extent [4]. Compared to the CK group, the pH of the T1 group remained largely unchanged, indicating that BC addition had minimal impact on soil properties and did not significantly contribute to soil salinization. The decrease in pH observed in the T6 group may be attributed to the precipitation of iron ions in nZVI@BC, which subsequently combined with OH^−^ after hydrolysis, leading to a decrease in pH [32]. The CEC value of the T6 group increased by 26.72 cmol^+^/kg, indicating that nZVI@ BC can improve soil fertility to a certain extent. Alpha diversity indices reflect the richness and diversity of microbial communities, which play a key role in maintaining ecosystem productivity, functional stability, and resilience against external pressures and disturbances [33]. The Chao1 index provides insights into the richness of microbial communities, while the Shannon index assesses the degree of microbial diversity. An increase in Pielou’s evenness index indicates a more equitable distribution within the community. The observed decrease in Chao1 and Shannon values in the soil following the addition of BC and nZVI@BC may be attributed to the alteration of the soil environment by biochar, which some of the microorganisms were unable to adapt to in the short term, leading to a decrease in their populations. To further evaluate the impacts of nZVI@BC on the diversity and abundance of microbial communities in Cr-contaminated soils over an extended period, the relative abundance of microorganisms at the phylum, order, and genus levels was analyzed. At the phylum level, Proteobacteria exhibited the highest relative abundance among all treatment groups, consistent with previous studies on metal-resistant bacteria [34]. The increased relative abundance of Alphaproteobacteria, Gammaproteobacteria, and Bacteroidia indicated that the addition of nZVI@BC created favorable conditions for bacterial colonization [35]. At the genus level, the T6 group demonstrated the highest microbial diversity index, indicating that nZVI@BC alleviated the environmental stress induced by Cr on soil microorganisms and contributed to the restoration of soil microbial diversity [36]. The Venn diagram illustrates the distribution differences and structural changes in communities across various treatments. In Cr-contaminated soil, 299 microorganisms co-existed across the three treatment groups, with each group also developing its own unique strains post-treatment [37]. Notably, the number of OTUs was significantly higher in the nZVI@BC treatment compared to the BC group, further indicating that nZVI@BC supports the enhancement of biodiversity in Cr-contaminated soils. In summary, nZVI@BC effectively remediates chromium-contaminated soil, improves the richness and diversity of microorganisms, and regulates the structure of these communities.

## 5. Conclusions

Nano zero-valent iron loaded by biochar was prepared using a bioleaching co-pyrolysis method in this study. The mechanisms of iron loading onto biochar during bioleaching and the iron transformation mechanism during co-pyrolysis were investigated. Furthermore, the structural characteristics and physicochemical properties of nZVI@BC were studied using modern chemical methods and instrumental analyses. The effectiveness of nZVI@BC was tested in experiments aimed at Cr removal in both water and soil. The results show that nZVI@BC demonstrates a promising capacity for Cr removal in water when compared to BC, BBC, and PBC-1000, with the removal efficiency ranking as follows: nZVI@BC > PBC-1000 > BBC > BC. Additionally, nZVI@BC effectively remediates Cr-contaminated soil, where Cr removal is enhanced by the application of nZVI@BC. The fixation rate of Cr in soil showed a positive correlation with the application of nZVI@BC. Moreover, the addition of nZVI@BC improved several physical and chemical properties of the soil, reducing the likelihood of salinization, enhancing the richness and diversity of the microbial community, and improving the growth and reproduction of indigenous bacteria to a certain extent. The nZVI@BC removes Cr(Ⅵ) from the water through mechanisms such as adsorption, reduction, and co-precipitation. In the case of Cr-contaminated soils, the solidification mechanisms mainly involve surface adsorption, electrostatic attraction, ion exchange and redox, and complexation and co-precipitation.

## Data Availability

The data presented in this study are available on request from the corresponding author.

## Supplementary Materials

The following supporting information can be downloaded at: https://www.mdpi.com/article/10.3390/nano14231895/s1, Table S1 Primer information for sequencing samples; Table S2 Percentage of atmosphere in tube furnace for different pyrolysis feedstocks at 1000 °C; Table S3 Specific surface area and pore structure of BC, BBC, nZVI@BC; Table S4 Kinetic modelling parameters for Cr (VI) adsorption by nZVI@BC; Table S5 Alpha diversity index table for chromium contaminated soils; Figure S1 SEM of BBC (A), GT map of BBC (B); Figure S2 SEM of PBC600, PBC800 (A, C) and particle size distribution calculation (B, D); Figure S3 Absorption and desorption isotherms and pore size distributions of nZVI@BC; Figure S4 XPS plenary spectra (A) and Cr 2p (B), C1s (C), O1s (D), Fe 2p (E) XPS fine spectra before and after the reaction of Cr adsorption by nZVI@BC; Figure S5 Effect of pH (A), dosage (B), initial concentration (C), temperature (D), and interfering ions (E) on the removal of Cr(Ⅵ) by nZVI@BC, and zeta potential of nZVI@BC (F); Figure S6 Kinetics of Cr(VI) adsorption by nZVI@BC: proposed primary model (A), proposed secondary model (B).
