# Supplementary material for: Mechanisms of Chromium Removal from Water and Soil Using Bioleached Nano Zero-Valent Iron-Mediated Biochar via Co-Pyrolysis
Source: Nanomaterials (Basel). 2024 Nov 26;14(23):1895. doi: 10.3390/nano14231895 (PMC11643807; doi:10.3390/nano14231895)
Supplement: Supplementary file 1 [file nanomaterials-14-01895-s001.zip › nanomaterials-3305156-supplementary.pdf]

Table S1 Primer information for sequencing samples

Name	Sequence (5'-3')	Types
PbT	AGCGCGCCCAGGAGCGCAGCGTCTT	Forward Primer
	GGC TCG AAG CCG TCG AGR TA	Reverse Primer
NitR	GACACCCGCCCGCATCTCAT	Forward Primer
	TGTCCCAGTCGCCTTCCACC	Reverse Primer
ChrA	GAATGCGCCCATGAAACC	Forward Primer
	TAGCACCTGTCGTTCTGTT	Reverse Primer

Table S2 Percentage of atmosphere in tube furnace for different pyrolysis feedstocks at 1000°C

Gas type / pyrolytic material	H_2	N_2	CO	CH_4	CO_2
BBC	14.07%	1.03%	29.44%	6.97%	48.47%
BBC + Sawdust	22.15%	4.24%	51.42%	0.89%	21.28%

Table S3 Specific surface area and pore structure of BC, BBC, nZVI@BC

Sample	Surface area/($m^2 \cdot g^{-1}$)	Pore volume/($cm^3 \cdot g^{-1}$)	Average pore diameter / nm
BC	41.09	0.021	3.44
BBC	52.74	0.056	4.83
nZVI@BC	318.10	0.073	4.69

Table S4 Kinetic modelling parameters for Cr (VI) adsorption by nZVI@BC

Absorbent	Proposed first-level kinetic model			Proposed secondary kinetic model		
	K_1 min^{-1}	q_e mg g^{-1}	R^2	K_2 g (mg $min)^{-1}$	q_e mg g^{-1}	R^2
BC	-0.0663	9.541	0.9505	0.3214	11.23	0.9709
BBC	-0.0748	10.36	0.9599	0.2434	12.66	0.9932
PBC	-0.0970	18.31	0.9562	0.0841	21.22	0.9996
nZVI@BC	-0.0979	29.32	0.9574	0.0560	32.89	0.9998

Table S5 Alpha diversity index table for chromium contaminated soils

Sample	Chao1	Observed_species	Pielou's evenness	Shannon	Simpson
CK	3746	3219	0.7691	8.91	0.990
BC	1429	1302	0.6511	6.78	0.960
T6	1763	1542	0.6618	7.09	0.975

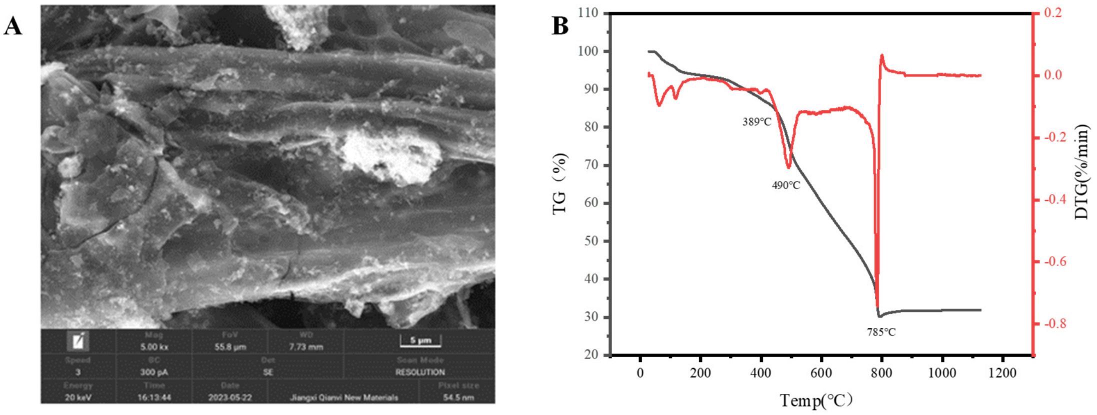

Figure S1 SEM of BBC (A), GT map of BBC (B)

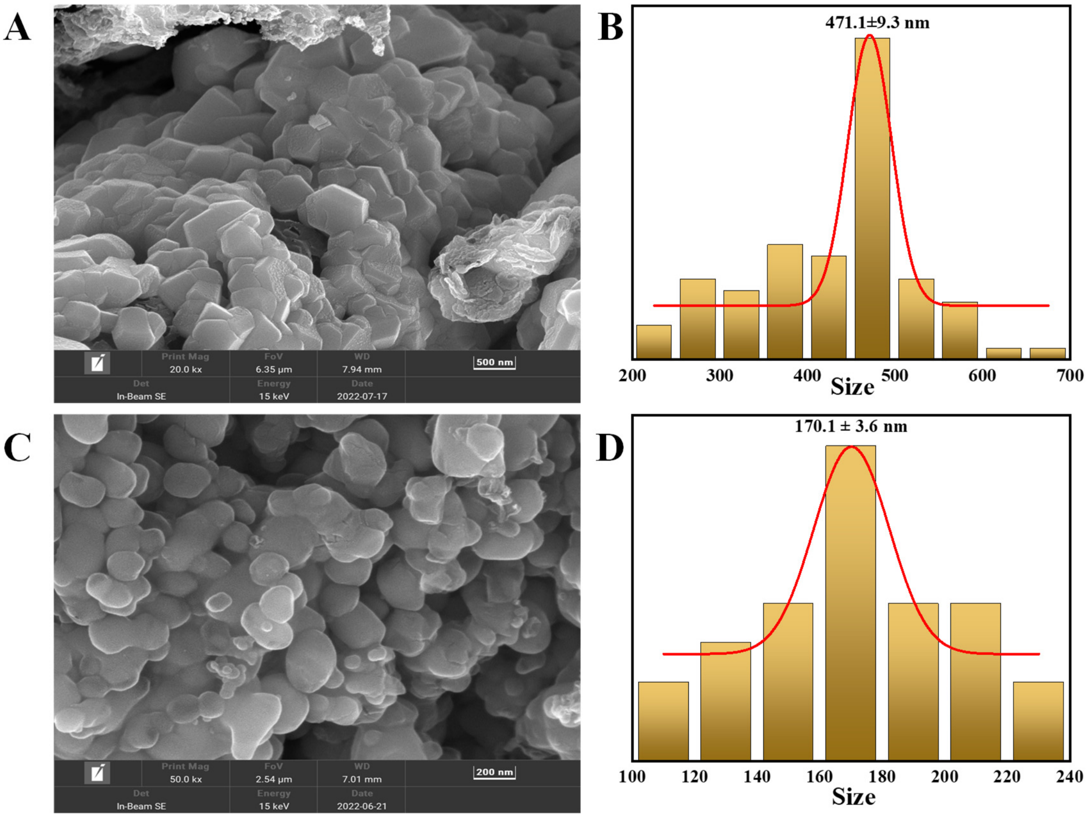

Figure S2 SEM of PBC600, PBC800 (A, C) and particle size distribution calculation (B, D)

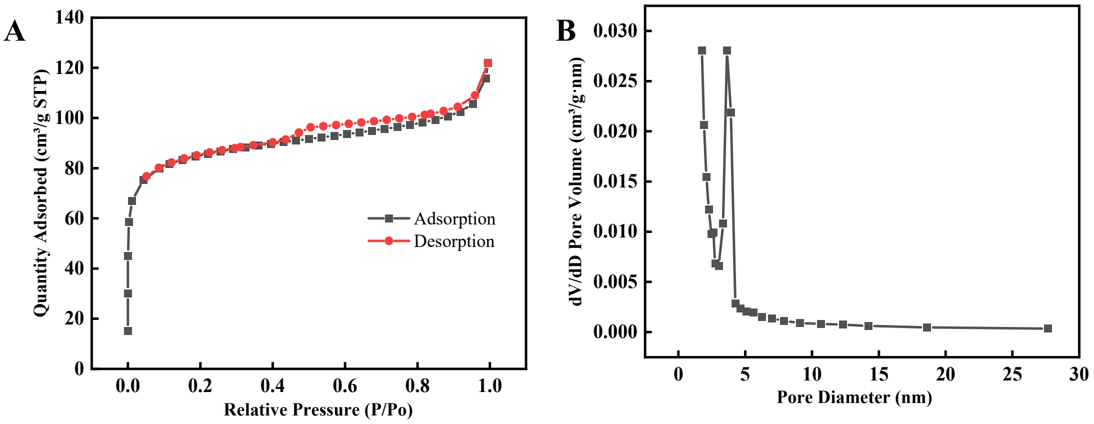

Figure S3 Absorption and desorption isotherms and pore size distributions of nZVI@BC

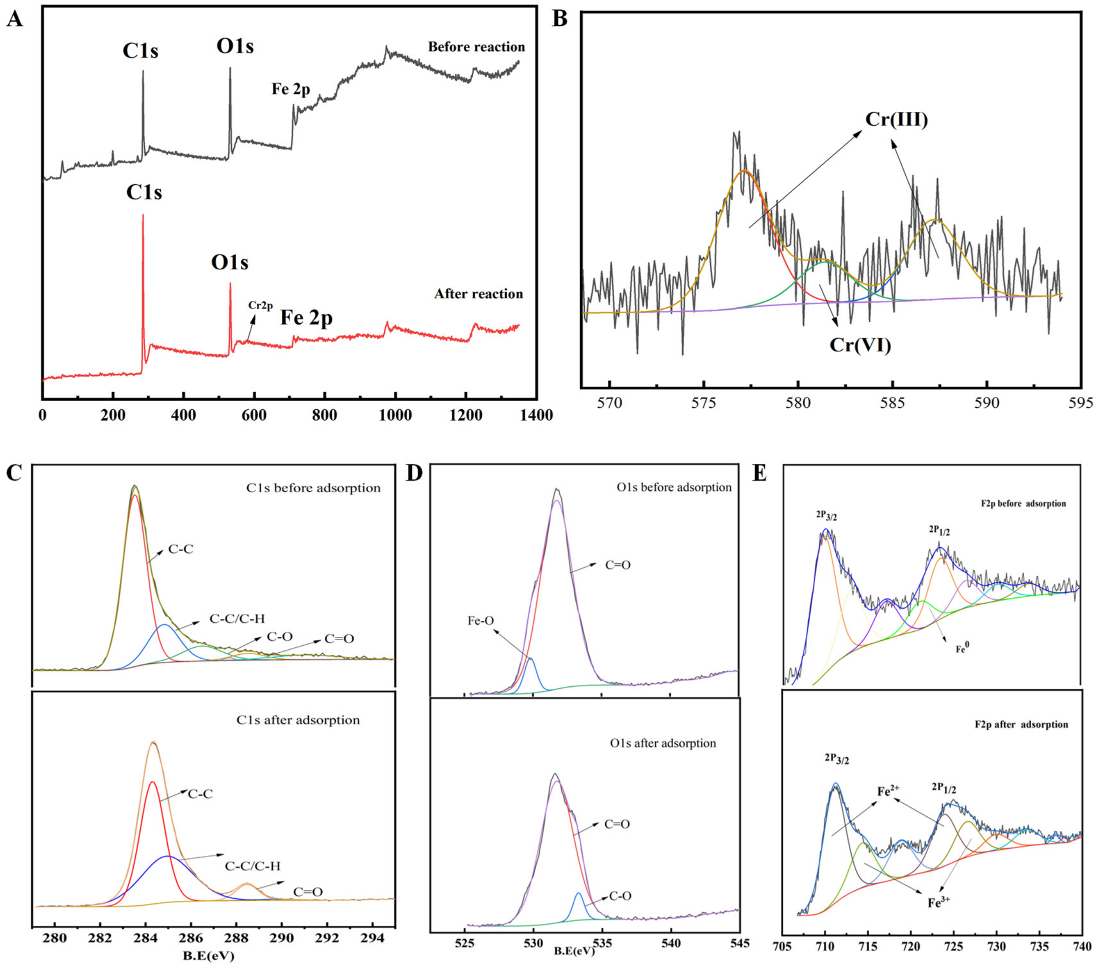

Fig. S4 XPS plenary spectra (A) and Cr 2p (B), C1s (C), O1s (D), Fe 2p (E) XPS fine spectra before and after the reaction of Cr adsorption by nZVI@BC

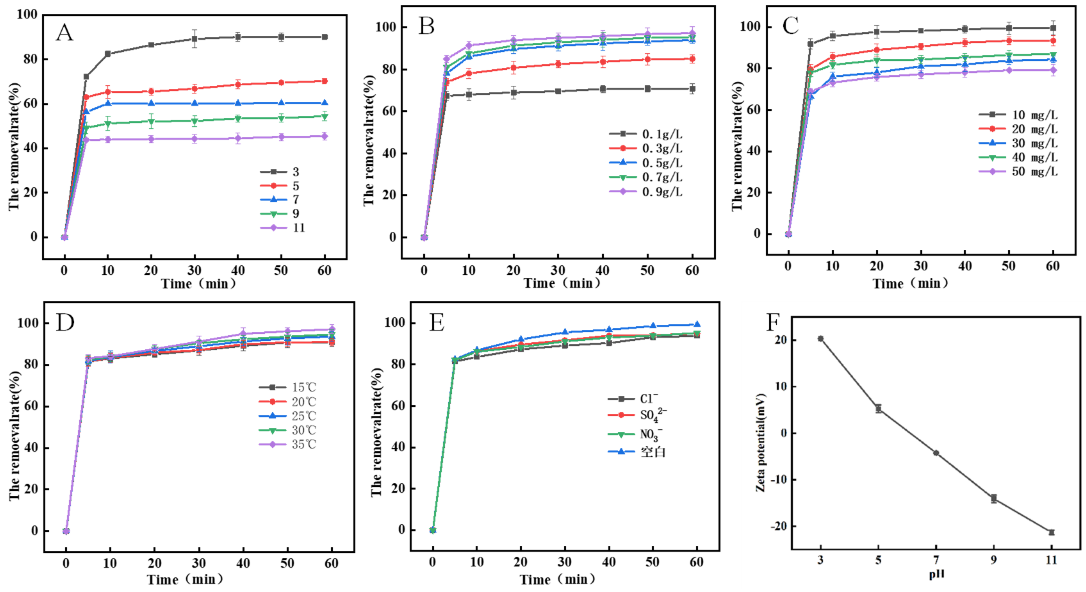

Figure S5 Effect of pH (A), dosage (B), initial concentration (C), temperature (D), and interfering ions (E) on the removal of Cr(VI) by nZVI@BC, and zeta potential of nZVI@BC (F)

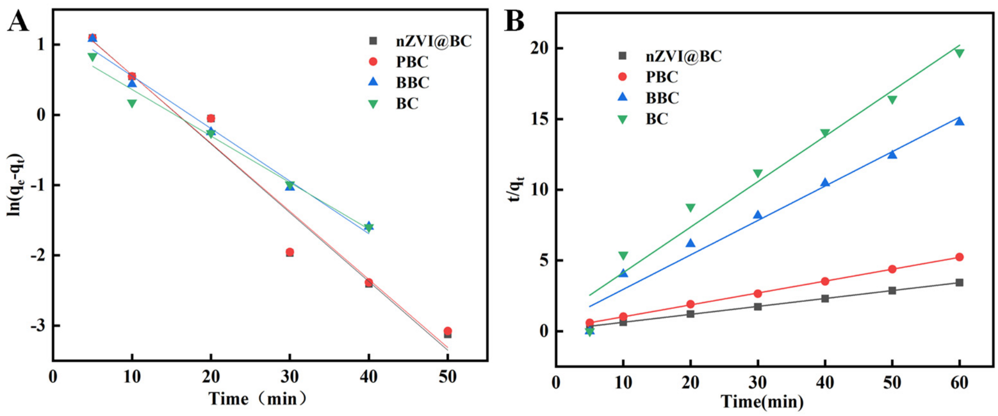

Figure S6 Kinetics of Cr(VI) adsorption by nZVI@BC: proposed primary model (A), proposed secondary model (B)
